# The Principles of Animal and Vegetable Physiology

**Published:** 1854-10

**Authors:** 


*The Principles of Animal and Vegetable physiology; A popular Treatise on the functions and phenomena of Organic Life. To which is prefixed a General View of the great departments of human knowledge.* By G. STEPHENSON BUSHNAN, M. D., Physician to the Metropolitan Free Hospital, &c. &c., with 102 illustrations on wood. Blanchard and Lea, Philadelphia, 1854.

This is a re-print from the London edition, of an Octavo volume of 234 pages. It is composed of two parts. The first is "on the nature, connexions and uses of the great department of human knowledge." The second is on "the Physiology of Animal and Vegetable Life." The book is designed, not for *professional*, but for *popular* reading and study. It is written in good style, and though necessarily brief in the discussion of the great science of Physiology, it presents that science in its true state, the author being well versed in the recent improvements which have been made in the branch of knowledge on which he writes. As a popular treatise for the general reader, it is doubtless one of the best now before the public.

